# Understanding how the informed consent process influences the decision to participate in an adaptive platform trial: A scoping review

**DOI:** 10.1371/journal.pone.0344560

**Published:** 2026-04-22

**Authors:** Nicole Yada, Luma Samawi, Neill K.J. Adhikari, Jennifer L. Gibson, Robert J. Reid, Robert A. Fowler

**Affiliations:** 1 Institute of Health Policy, Management and Evaluation, Dalla Lana School of Public Health, University of Toronto, Toronto, Canada; 2 Unity Health Toronto, Toronto, Canada; 3 Department of Critical Care Medicine, Sunnybrook Health Sciences Centre, Toronto, Canada; 4 Joint Centre for Bioethics, Dalla Lana School of Public Health, University of Toronto, Toronto, Canada; 5 Trillium Health Partners, Institute of Better Health, Mississauga, Canada; University of Texas Southwestern Medical Center at Dallas, UNITED STATES OF AMERICA

## Abstract

**Introduction:**

The complex design of adaptive platform trials may be challenging for prospective participants and their families and/or substitute decision makers to understand when approached for informed consent. The purpose of this scoping review is to explore and synthesize literature on how the informed consent process influences the decision to participate in an adaptive platform trial.

**Methods:**

A scoping review of empirical literature published in English was conducted using MEDLINE, EMBASE and PsychInfo databases (1976–2025). A key term search strategy examined the overarching concepts of informed consent process and adaptive platform trials. Empirical studies whose participants were adults in a health care delivery or clinical research setting were included. The outcome of interest was factors that affect eligible trial participant decisions to participate in adaptive platform trials. Grey literature was further searched to substantiate and complement the peer reviewed literature.

**Results:**

Of 1085 articles reviewed, four met inclusion criteria. The four papers highlighted challenges in explaining to prospective participants how the randomization ratio may change during the trial and how likely participants are to benefit, as well as the importance of communication outside of the written consent form. Eight grey literature results were included, covering an emerging policy and regulatory discourse, as well as nascent empirical studies examining alternate delivery modalities for consent information.

**Conclusions:**

Limited data highlight the importance of empirical investigation into how to facilitate informed participation in adaptive platform trials. More research is needed to understand how this trial design may affect enrollment decisions and therefore associated policies and processes needed to support informed participation in this modernized trial design.

## Introduction

**Fig 1:**
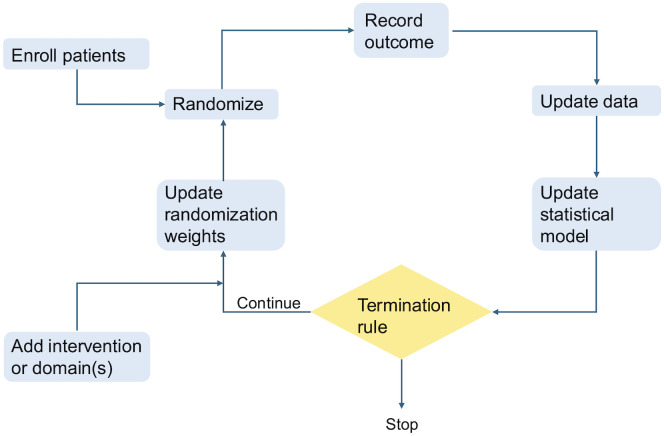
General operational flow of an adaptive platform trial. As appears in: The Adaptive Platform Trials Coalition. Adaptive platform trials: definition, design, conduct and reporting considerations. Nat Rev Drug Discov 18, 797–807 (2019). Reproduced with permission from SNCSC.

Researchers, funders and policymakers are embracing the concept of conducting clinical research as an integrated feature of the healthcare system to increase research efficiency and timely translation of knowledge into practice [1–3]. One such way to do so is through adaptive platform trials, which are a type of clinical trial design [4]. The “adaptive” component refers to using accumulating data from trial participants to make prospectively planned modifications to one or more aspects of the design [5]. The “platform” component refers to the durable, long-term mechanism to evaluate multiple interventions. Trials can be based on platforms, and not be adaptive, or adaptive without being in a platform. Brought together, this new type of trial design has gained widespread popularity in a brief period of time. See Fig 1 for a diagram of the general operational flow of an adaptive platform trial [6].

In these types of trials, with randomization decisions favouring the intervention showing the most promise, there is a greater likelihood that the interventions under evaluation may be of clinical benefit to research participants [7] – challenging traditional notions of who directly benefits, and when, from research. Large adaptive platform trials embedded into health systems played a critical role generating evidence about therapeutics for pandemic coronavirus disease [8], and are increasingly used in a broad range of care [9–11].

Traditional randomized controlled trials focus on studying a *specific* trial intervention for a disease or condition in trial bounded by a fixed sample size, whereas adaptive platform trials focus on a disease or condition, and study many trial interventions in parallel, the interventions often changing over a longer period and without fixed sample sizes. The intent of the adaptive trial design is to operate continuously as a platform and facilitate adaptive comparisons of alternate study or comparator interventions. In an adaptive platform trial, consented and enrolled participants generate data that iteratively update a pre-specified algorithm model, which uses decision rules to end use of a specific intervention found to be of sufficiently low probability of effectiveness. A common adaptive component in this trial design is the use of response adaptive randomization (RAR), whereby randomization instructions are updated to steer randomization towards trial interventions that have a higher chance of effectiveness, but that require more evidence and greater certainty [4,6]. Some have argued that the participant in an adaptive platform trial *may* be safer participating than not – increasing odds of benefit runs countenance to traditional trials for which equipoise must be present [7]. Others have cautioned that there can be a loss of statistical efficiency when randomization allocation is not 1:1 [12]. Faster accrual and translation of research knowledge into clinical practice to drive care improvement is one demonstration of how a healthcare system can learn from itself – or more specifically, from conducting such trials – more efficiently than ever before [6]. As these trials typically occur in real-world settings such as hospitals, often rely on standard of care for comparator arms and aim collect data in near-real time, they represent an example of the conceptualization of clinical research as *part of* usual care [6,7].

Participants have been found to enroll in trials for a variety of reasons, including seeking therapeutic benefit [13–15], and for altruistic purposes [15–17]. The phenomenon of trial participants confusing clinical trial interventions with individualized care is known as the therapeutic misconception [18]. While therapeutic misconception has traditionally been viewed as something to avoid when designing and implementing studies, given this design’s deliberate integration into care delivery described above, adaptive platform trials challenge the need for individuals to dichotomize research participation and the care they receive.

Informed consent is a required ethical component of clinical research and relies on participants receiving information about potential study risks and benefits and using that information to make decisions about participation [19–21]. Prior research has found that decisions to participate in research are a product of a variety of factors that exist outside of the written informed consent decision, such as relationship with the care/research team and understanding of how risks and benefits applied to them [22–26]. Further, prior research that has cited the importance of attributes such as good communication skills, approachability, trustworthiness, person-centredness and being knowledgeable, among those delivering trial information to support participant enrollment [15]. Inherent in the design of adaptive platform trials is that treatments offered are often numerous, and in addition, they change over the course of a trial [27]. In a setting such as the intensive care unit or emergency room, the timeframe for enrollment is often short – adding an additional time pressure to the encounter [28,29]. Given prior research on challenges with the length and readability of consent forms for traditional clinical trials [20,30] it is possible that the complex and longitudinal design of adaptive platform trials may be challenging for prospective participants and their families/substitute decision makers to understand when they are approached for consent. Further, adaptive platform trials are not meaningfully described or mentioned in ethical and regulatory policies for human subjects research [31,32], resulting in minimal guidance for researchers and ethics practitioners when designing ethics documentation, such as informed consent forms, for these types of trials. To date, little is known about how this trial design affects the decision to enroll in the study, if at all.

The objective of this paper is to explore and synthesize literature on what is known about **how the informed consent process influences the decision to participate in an adaptive platform trial.** The term “consent process” will be used throughout this paper to encompass how information is delivered, understood, and used to make research participation decisions, which may include refusal of participation and withdrawal of consent during the study.

## Methods

The choice to approach this topic through a scoping review is due to the increasing popularity of adaptive trial designs yet limited ethical guidance on their conduct. Scoping reviews are appropriate when an overview of the topic or phenomena at hand is required before more specific, targeted questions can be asked [33].

Preliminary and background searches were conducted to get an initial sense of the scope and type of existing studies on concepts of interest. Population, concept, and context were used to identify inclusion and exclusion criteria. Empirical studies (quantitative, qualitative and mixed methods) whose participants were adults (18 years of age and older) in a health care delivery and/or clinical research setting, as patients, research participants or those working these areas and engaged with the consent process were included. See Table 1 for full inclusion and exclusion criteria. The search strategy was developed in consultation with a medical librarian and in compliance with the Peer Review of Electronic Search Strategies (PRESS) reporting guidelines to evaluate the quality of the search strategy [34]; these guidelines complement the Preferred Reporting Items for Systematic reviews and Meta-Analyses extension for Scoping Reviews (PRISMA-ScR) guidance [35] also used and described in this manuscript.

**Table 1:** Scoping review search strategy for peer-reviewed publications.

What is known about how the informed consent process influences the decision to participate in an adaptive platform trial?	
**Search terms**	
**Concept 1: Informed consent process** *How information is delivered, understood, and used to make research participation decisions*	**Concept 2: Adaptive and/or platform trial**
exp Informed Consent/ Patient Education as Topic/ Communication/ informed decision making.kw,tw,kf. informed consent.kw,tw,kf. opt-in opt-out.kw,tw,kf. patient choice.kw,tw,kf. (benefits adj3 harms).kw,tw,kf. (risks adj3 benefits).kw,tw,kf. Patient Selection/es [Ethics] exp Risk Assessment/es [Ethics] Choice Behavior/ Decision Making/ Consent.kw,tw,kf. ethics/ research ethics/	Adaptive Clinical Trials as Topic/ exp Adaptive Clinical Trial/ (adapt* adj3 trial*).kw,tw,kf. adaptive clinical trial*.kw,tw,kf. (platform* adj3 trial*).kw,tw,kf. Adapt* design*.kw,tw,kf.
Databases	MEDLINE, EMBASE, PsycInfo
**Inclusion**	Population: Adults 18+ in a healthcare delivery and/or clinical research setting (as patients, participants or those working in these areas and engaged with the consent process) Topic: Communication of information about research participation (i.e., through informed consent processes) by clinicians and/or researchers to participants Context: Clinical research (human); English articles, Empirical (quantitative, qualitative, mixed methods) papers Outcome: Factors that affect eligible trial participant decisions to participate in adaptive and/or platform trials
**Exclusion**	Focus is on the ethics of the study topic (e.g., reproductive rights and medical assistance in dying) Articles focused on assessing capacity to consent (e.g., due to mental illness, incapacitation, end of life or psychiatric illness) Incentives to trialist (i.e., industry sponsorship) Macro-level topics (e.g., benefit sharing in a global health context)
**Timeframe**	Studies published from each respective database’s start dates until January 2025.

MEDLINE, EMBASE and PsycInfo databases were searched in January 2025, from each respective database’s start dates, with the earliest mention of adaptive trial designs in 1976 [36]. The overarching concepts of informed consent process and adaptive and/or platform trials were used. In initiating the search, keywords and where possible, controlled vocabularies such as Medical Subject Headings, were searched. Terms searched in MEDLINE included: Informed Consent, Patient Education as Topic, Communication, informed decision making, informed consent, opt-in opt-out, patient choice, benefits adj3 harms, risks adj3 benefits, Patient Selection [Ethics], Risk Assessment [Ethics], Choice Behavior, Decision Making, Consent, ethics, research ethics, Adaptive Clinical Trials as Topic, adapt* adj3 trial*, adaptive clinical trial, platform* adj3 trial*, adapt* design*. The above search terms were adapted to spelling variations or hyphenation. This search returned 602 results. This same search strategy was adapted and repeated in the other databases and yielded an additional 699 results; 216 duplicates were removed. The search strategies for each database can be found in S1 Text – Sections 1–3.

Relevant references were hand searched; the search was limited to English articles and research involving humans. Titles and abstracts were double screened by NY and LS. NY and LS met regularly to resolve conflicts as they arose. NY and LS did full text screening of all citations that met selection criteria. Screening was conducted independently using Covidence [37]. Following eligibility screening, data were abstracted by NY and LS for each of the final papers selected. A form to chart the data was developed to identify the fields of:

1. Author, year
2. Study origin
3. Aims/purpose
4. Study population and sample size
5. Methodology/methods
6. Key findings

To present an overview of literature findings, results are summarized in both in Table A in S1 Table and through a narrative overview of themes identified [38]. Due to the limited peer reviewed studies, we reviewed the grey literature using a structured search process [39] to contextualize the empirical results and understand the emerging discourse in which this trial design is being used. The grey literature search was intended to elicit inclusive results, and as such the first 100 results were screened using the same search concepts above (informed consent process; adaptive and/or platform trial). The following fields were extracted from the grey literature: 1) Author, 2) Year, 3) Paper origin, 4) Description and 5) Key findings. Results were double-screened, extracted and presented in Table B in S1 Table.

## Results

### Included studies

**Fig 2:**
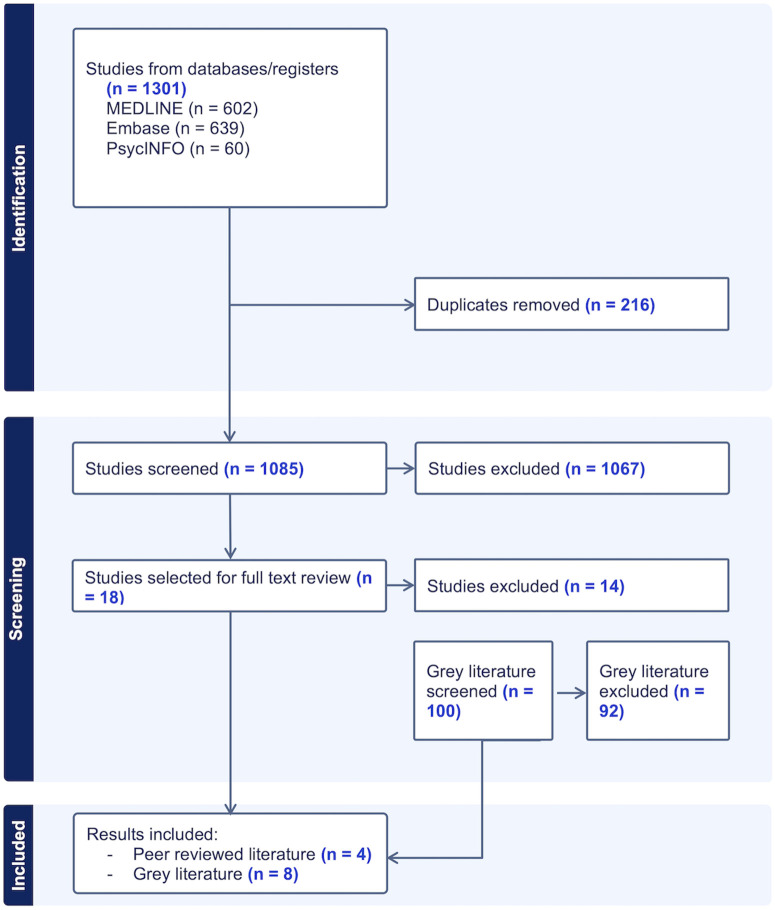
PRISMA diagram of studies identified, screened, and included.

Across the three databases, the search returned 1085 articles, of which 4 were deemed eligible. Of these, two studies included prospective trial participants [40,41], another included a patient advisory panel and a central institutional review board [42], and another included researchers from bioethics, epidemiology, biostatistics and/or medical backgrounds [43]. All studies originated from the United States or Europe. Three studies were qualitative, using interviews and/or focus groups [40,42,43] and the remaining paper collected quantitative data via survey [41]. See Fig 2 for a PRISMA flow diagram of study selection; the PRISMA-ScR checklist is included as S2 File.

### Describing adaptive randomization and likelihood of benefit in the consent form

All studies examined how to describe that randomization would adapt over time during the consent process. One study included a statement in the consent form about how randomization would adapt [40], another did not, citing concerns about overstating the likelihood of benefit [42]. Tehranisa and Meurer (2014) specifically examined whether participants would be more likely to enroll in a hypothetical study that included response adaptive randomization (RAR), and found that this did significantly increase enrollment by 12.8% compared to the standard trial [41]. While both the control group (those whose consent form did not include RAR) and intervention group had similar self-reported understanding of the study design, significantly fewer in the RAR group correctly identified how trial allocation would occur [41].

Ways to frame individual benefit in the consent form was raised in all four studies, for example, that one is more likely to receive a promising intervention the later they enroll in the study and that the design of an adaptive platform trial means that the prospective participant is – on average, safer participating than not. Dickert et al. (2022) worked with a research ethics board (REB) and patient advisory panel to design a consent process for a multicenter trial incorporating adaptive randomization [42]. While the patient advisors originally believed that *not* disclosing full details about how randomization worked would compromise participant autonomy, upon further discussion with the REB, the patient advisors elected to add a new statement to the consent (“It is possible but unknown whether adding these medicines will help reduce the impact of your stroke”) [42]. The patient advisors ultimately decided to not describe RAR due to concern over the time it may take to describe and so as not to overstate the likelihood of individual benefit. In their description of RAR in the consent form, Tehranisa and Meurer (2014) noted that depending on the intervention effectiveness, participants had up to a 80:20 likelihood of receiving the better performing treatment [41]. Symons et al. (2022) examined how patients and their caregivers viewed a layered (i.e., simplified) approach to consent, in which the key information for decision-making was provided in a shorter informed consent form with additional optional information that is accessed separately. They further elicited input on the optimal content and layout of the layered consent materials for a large adaptive platform trial [40]. Participants elected to include the following statement in the consent form: “This study is called an Adaptive Platform Trial. In this type of study, the researchers analyse the results as the study goes on rather than just at the end. This means that people who take part in the study once it has been running for a while have a better chance of getting a better treatment” [40]. Participants in this study suggested that any description of potential benefit should be included near the start of the consent form as it would affect their participation decisions [40]. While most included studies noted the importance of not overstating benefits to avoid therapeutic misconception, all four studies used the term “treatment” to refer to the study intervention.

### The role of communication between the research team and trial participants in the consent process

Three papers described the importance of communication outside of the content of the consent form [40,42,43]. Chongwe et al. (2020) found that while the design of adaptive platform trials is complex and thus challenging to describe, this complexity is not an insurmountable challenge for adequately consenting participants to this type of study [42]. These papers recommend the use of varied communication modalities (e.g., use of multimedia) and plain language descriptions of study design concepts, and consideration of consent as an iterative process. Personal contact with the research team during the enrollment period was cited by Dickert et al. as important to provide comfort and clarification of complicated study aspects [42]. In this same study, while the patient advisors elected to not describe RAR in the consent form itself, they noted it would be helpful to have the investigator or research coordinator explain the process at a less-acute time than enrollment [42]. Three of the four papers raised the importance of using person-centered research approaches, such as designing trials that consider participant perspectives to facilitate more accessible and ethical involvement in adaptive platform trials [40,41,43]. Two of the included papers raised regulatory and practical considerations in adaptive platform trial design, such as how ethical frameworks must evolve to accommodate such innovative designs, and the need to balance scientific rigour with ethical imperatives to protect participants [43,42].

### Grey literature

A search of the grey literature returned an additional 100 results, of which 8 were deemed relevant to the topic of informed consent to adaptive platform trials and are described in Table B in S1 Table. A 2019 meeting report on ethical issues in adaptive and platform trial designs noted variable approaches used in consent forms in response to items specific to this trial design, such as adaptations to randomization or the addition or removal of trial arms may affect the amount – and timing – of information delivery to participants (i.e., explaining all possible permutations upfront in the form or reconsenting if/when information becomes relevant). The report called for the need for developing standards for informed consent documentation for adaptive platform trials to increase consistency in approach used, and clear guidance for how researchers explain adaptive designs to facilitate better understanding among REBs and participants [44]. In 2024, the World Health Organization (WHO) released their *Guidance for Best Practices for Clinical Trials* [8] in response to the 2022 World Health Assembly resolution WHA75.8 (2022) on strengthening clinical trials [45]. The Guidance noted a pressing need for promoting understanding and adoption of innovative trial designs, including adaptive platform designs. A 2023 WHO Meeting Report on ethics and adaptive platform trials in public health emergencies cited an absence of empirical research into what participants would find beneficial to know in deciding to participate in this type of trial, including whether there should be notification of added or removed arms during the course of the study to enrolled participants, and how to acknowledge uncertainties and accruing knowledge during the consent process [46]. The report called for dedicated research into these areas. In 2024, the Food and Drug Administration released draft guidance on Master Protocols (which include adaptive and platform trial designs). The guidance notes that the informed consent process and documentation should cover all treatment arms or domains to which the participant could be randomized, as introducing treatment arms after the initial consent could reduce comparability if study participants differ between those who would, for example, consent to participating in domain A from those that would consider enrolling in the domain B [47].

In a Canadian context, the Tri-Council Policy Statement: Ethical Conduct for Research Involving Humans (TCPS 2 2022) includes reference to adaptive trial designs, and notes the increased likelihood of benefit as the trial progresses and that adaptive randomization may prioritize participants towards interventions for which unknown side effects take longer to appear. TCPS 2 further notes that while adaptive trials may require fewer participants, this can limit opportunities for targeted sub-group analyses [31]. The grey literature search further identified three, Canadian-led protocols for nascent work underway to explore the effect of innovative methods for delivering information contained in the informed consent form for existing adaptive platform trials [29,48,49], but the focus is on format or modality of delivery, and not the specific language or ethical concepts to include.

## Discussion

A predominant finding from the empirical and grey literature is that evidence is needed to guide how the informed consent process can support participation in adaptive platform trials. Key themes identified were: that descriptions of adaptive randomization and likelihood of benefit in the consent process can affect how participants perceive enrollment in an adaptive platform trial, and that communication outside of the written consent form is especially necessary for this type of trial design.

The limited number of empirical studies found during this review highlights the need for additional investigation to understand what participants both want and need to know to support their informed participation in the increasingly popular adaptive platform trial study design. Future research should further examine responses in real-time settings as participant preferences for consent information may be different when faced with a “live” enrollment decision, especially if they have not previously participated in a trial [15]. Common across included results was the need for empirical investigation into whether and how to describe RAR in the consent process, and relatedly, the implications for participant benefit by being part of the trial. Both the amount of information and the content itself should be designed to strengthen participant autonomy in their decision-making.

From a practical standpoint, the way consent forms are structured to reflect the specific elements of adaptive platform trials (including RAR and the use of multiple, evolving study domains) warrants further investigation. Aspects unique to adaptive platform trials that may affect participation decisions to enroll in the study include consideration of whether study methods and benefits (and risks) should be viewed distinctly, given RAR can affect the participant’s likelihood of benefit, and whether all possible study domains should be described at the study outset or introduced separately as they become relevant.

### Limitations

This review included only empirical studies and grey literature. A substantial amount of the ethics literature included in the initial screening results were theory or comment papers. It is therefore possible that insights noted above about the lack of ethical foundation in how studies were framed may have been noted in these excluded studies. Double screening of both empirical and grey literature was conducted to further substantiate the findings and mitigate the potential for missing data. The increasing use of adaptive platform trials means a rapidly changing evidence base, necessitating periodic updates through a refresh of the search results.

## Conclusions

Adaptive platform trials allow for learning from each person that enters a study and using that information to inform the study’s ongoing conduct. In doing so, participants can receive the most promising interventions – and evidence is generated to improve how care is delivered.

This scoping review sought to understand what is known about how the informed consent process influences the decision to participate in an adaptive platform trial. The limited results found through this review highlight the importance of dedicated empirical investigation into how to facilitate informed participation into this type of trial design. A predominant finding from the included empirical and grey literature is that evidence and guidance are needed to support participants with this type of trial design. The ethical nuances introduced by adaptive platform trials are well documented, but the policy and practice implications much less so. This paper serves as a call to action to develop associated policies and processes that support informed participation in the modernized view of the interplay between research and care that adaptive platform trials provide.

## Supporting information

S1 Text (DOCX)

S1 Table (DOCX)

S1 File (CSV)

S2 File (DOCX)
