# Supplementary material for: Understanding how the informed consent process influences the decision to participate in an adaptive platform trial: A scoping review
Source: PLoS One. 2026 Apr 22;21(4):e0344560. doi: 10.1371/journal.pone.0344560 (PMC13102233; doi:10.1371/journal.pone.0344560)
Supplement: S1 Text — (DOCX) [file pone.0344560.s001.docx]

## S1 Text - Supporting Information

1. Ovid MEDLINE Search Terms
2. Embase Search Terms
3. APA PsycInfo Search Terms

## Section 1: Ovid MEDLINE Search Terms

1. exp Informed Consent/

2. Patient Education as Topic/

3. Communication/

4. informed decision making.kw,tw,kf.

5. informed consent.kw,tw,kf.

6. opt-in opt-out.kw,tw,kf.

7. patient choice.kw,tw,kf.

8. (benefits adj3 harms).kw,tw,kf.

9. (risks adj3 benefits).kw,tw,kf.

10. Patient Selection/es [Ethics]

11. exp Risk Assessment/es [Ethics]

12. Choice Behavior/

13. Decision Making/

14. Consent.kw,tw,kf.

15. ethics/

16. research ethics/

17. Adaptive Clinical Trials as Topic/

18. exp Adaptive Clinical Trial/

19. (adapt* adj3 trial*).kw,tw,kf.

20. adaptive clinical trial*.kw,tw,kf.

21. (platform* adj3 trial*).kw,tw,kf.

22. Adapt* design*.kw,tw,kf.

23. 1 or 2 or 3 or 4 or 5 or 6 or 7 or 8 or 9 or 10 or 11 or 12 or 13 or 14 or 15 or 16

24. 17 or 18 or 19 or 20 or 21 or 22

25. 23 and 24

## Section 2: Embase Search Terms

1. exp Informed Consent/

2. Patient education/

3. Interpersonal Communication/

4. informed decision making.kw,tw,kf.

5. informed consent.kw,tw,kf.

6. opt-in opt-out.kw,tw,kf.

7. patient choice.kw,tw,kf.

8. (benefits adj3 harms).kw,tw,kf.

9. (risks adj3 benefits).kw,tw,kf.

10. Patient Selection/

11. Risk Assessment/

12. Choice Behavior.kw,tw,kf.

13. *Decision Making/

14. Consent.kw,tw,kf.

15. ethics/

16. research ethics/

17. Adaptive Clinical Trials as Topic/

18. exp Adaptive Clinical Trial/

19. (adapt* adj3 trial*).kw,tw,kf.

20. adaptive clinical trial*.kw,tw,kf.

21. (platform* adj3 trial*).kw,tw,kf.

22. Adapt* design*.kw,tw,kf.

23. 1 or 2 or 3 or 4 or 5 or 6 or 7 or 8 or 9 or 10 or 11 or 12 or 13 or 14 or 15 or 16

24. 17 or 18 or 19 or 20 or 21 or 22

25. 23 and 24

## Section 3: APA PsycInfo Search Terms

1. Informed consent/

2. Patient education/

3. Interpersonal communication/

4. Patient selection/

5. risk assessment/

6. Decision making/

7. informed decision making.tw.

8. informed consent.tw.

9. opt-in opt-out.tw.

10. patient choice.tw.

11. choice behavior.tw.

12. consent.tw.

13. ethics/

14. research ethics/

15. (benefits adj3 harms).tw.

16. (risks adj3 benefits).tw.

17. adaptive clinical trial*.mp.

18. (adapt* adj3 trial*).mp.

19. (platform adj3 trial*).mp.

20. adapt* design*.mp.

21. 1 or 2 or 3 or 4 or 5 or 6 or 7 or 8 or 9 or 10 or 11 or 12 or 13 or 14

22. 17 or 18 or 19 or 20

23 21 and 22
