# Supplementary material for: Understanding how the informed consent process influences the decision to participate in an adaptive platform trial: A scoping review
Source: PLoS One. 2026 Apr 22;21(4):e0344560. doi: 10.1371/journal.pone.0344560 (PMC13102233; doi:10.1371/journal.pone.0344560)
Supplement: S1 Table — (DOCX) [file pone.0344560.s002.docx]

## S1 Table - Supporting Information

1. Data Summary Table – Peer Reviewed Literature
2. Data Summary Table – Grey Literature

## Table A: Peer Reviewed Literature Data Summary Table

| **First author** | **Year** | **Study origin** | **Aims/purpose** | **Study population and sample size (if applicable)** | **Methodology/methods** | **Key findings that relate to research question(s)** |
| --- | --- | --- | --- | --- | --- | --- |
| Chongwe et al. (43) | 2023 | United States | To understand how methods and ethics experts believe adaptive trials affect the ethics of clinical research compared to traditional randomized controlled trials. | • Researchers from bioethics, epidemiology, biostatistics and/or medical backgrounds. • Eligible participants were those that had conducted an adaptive trial, had published a paper on adaptive designs or had served on an ethics committee where adaptive trials were reviewed. N=17. | In-depth, open-ended interviews were conducted. Snowball sampling was used to identify additional participants. The study participants were researchers involved in research in adaptive clinical trials, and were recruited from academic institutions in academic institutions and federal government agencies. | • As adaptive trials are more complex than ordinary clinical trials, there is a potential threat to the autonomy of participants should the study not be understandable.  • This threat to autonomy was viewed as surmountable; trial participants need not understand everything about the study for consent to be valid.  • Efficiency and potential for participant benefit were the main justification for use of the adaptive trial design.  • While some participants had concerns about the clinical equipoise being disrupted when response adaptive randomization updates participant allocation, others insisted that despite the changing the probabilities, uncertainty is still present, so clinical equipoise is not disrupted. |
| Dickert et al. (42) | 2022 | United States | To explore views regarding adaptive randomization and remote consent through individual and focus-group interviews with surrogates for participants who had been enrolled in prior stroke trials, and in collaboration with an existing patient advisory panel, to develop and implement a patient-centered consent process for the Multi-arm Optimization of Stroke Thrombolysis (MOST) trial. | • Individual interviews with former surrogate decision makers for participants in a prior study. N=6. • 2 focus groups with individuals who had served as surrogates in acute stroke trials. N=8. • Patient Advisory Panel of patients, surrogates, and patient-family advisors. N=7. • consultation with the central institutional review board. | Mixed-methods study involving focus groups and interviews, as well as collaboration with a patient advisory panel and a central institutional review board to design and implement a consent process for a multicenter trial that included adaptive randomization. | • Adaptive randomization viewed positively by participants, but the process of adaptive randomization requires substantial explanation and appeared prohibitively complex.  • Patient advisory members felt that it should be possible to explain the combination of potential for benefit and uncertainty about whether that benefit would happen, noting this is a basic element of understanding a clinical trial—a view consistent with other data demonstrating that information about potential for direct benefit is among the most important information to individuals considering participation.  • The following statement was included in the consent form after patients determined it was not necessary to describe response adaptive randomization during the initial consent process: “It is possible but unknown whether adding these medicines will help reduce the impact of your stroke.” |
| Symons et al. (40) | 2022 | England | The primary objectives were to gather participant views on: 1) The acceptability of a simplified consent form; 2) Whether the consent form contained sufficient information for decision-making; and 3) Whether inclusion of a "benefit statement" applicable to platform trials was relevant to a person’s risk–benefit assessment. | Adolescent and adult survivors of staphylococcus aureus bloodstream infection and their caregivers. N=24. | Qualitative multicentre study with focus groups and semi-structured interviews, involving | • Majority of participants supported the use of layered consent, whereby basic information is summarised upfront in the consent form, with embedded hyperlinks to supplemental information and multimedia for the trial, as felt it provided a greater sense of agency over the information to receive.  • They suggested that research consent presents a useful opportunity to improve knowledge about a potential trial participant’s disease condition.  • Participants also supported the inclusion of a statement in the consent form on benefits relevant to platform trials, as it was considered important for their decision-making. They also supported a more balanced description of the trial’s risk–benefit ratio, so that risks are not overstated. |
| Tehranisa& Meurer (41) | 2014 | United States | To understand how inclusion of response adaptive randomization in a hypothetical study affects enrollment to a trial. | Non-critically ill emergency department adult patients, without presenting symptoms consistent with stroke, altered mental status, or alcohol intoxication. N=418. | Cross-sectional randomized survey. Participants were randomly allocated to see one of two videos. They also answered questions about demographics and stroke symptom knowledge. The video was the same across both groups, with the exception of the explanation of the hypothetical study: either described as a standard clinical trial or a response-adaptive randomization study. All participants in both groups were informed that the trial had recruited approximately one half of the total planned enrollment and then were asked if they would participate in the stroke trial. | • The RAR trial design resulted in higher research participation rates than standard randomization for a hypothetical acute stroke trial (absolute increase of 12.8%).  • Self-reported understanding between the standard and response adaptive randomization (RAR) groups was not significantly different, however significantly fewer in the RAR group actually correctly identified the method of trial allocation.  • Implementing the RAR trial design could increase recruitment, and also the offer the overall trial population added benefit compared to a fixed randomization trial. Future studies should explore learning more about why RAR was preferred, or what specific components of RAR were viewed favorable, in order to understand how to optimize its implementation. Improving the rapid communication of adaptive clinical trial designs in settings such as the emergency room was further recommended as an area warranting further empirical investigation. |

## Table B: Grey Literature Data Summary Table

| **Author** | **Year** | **Paper origin** | **Description** | **Key findings** |
| --- | --- | --- | --- | --- |
| The Multi-Regional Clinical Trials Center of Brigham and Women’s Hospital and Harvard Bioethics Collaborative (44) | 2019 | USA | Meeting of the MRCT Center Bioethics Collaborative convened stakeholders from academia, industry, patient advocacy organizations, foundations, and Institutional Review Boards to examine the topic of ethical challenges in adaptive and platform trials. | Unresolved areas included:  • Which details of adaptive study design need to be disclosed and explained to research participants to obtain consent that is fully informed.  • Ideal document structure: Attendees agreed that the inapplicable information should be removed, and, ideally, the consent document should be tailored to each patient’s specific intervention. One attendee suggested a two-stage informed consent process for adaptive-platform trials: one consent form at the beginning of the trial that explains the general trial design, and a consent form after the participant is randomized to their specific intervention that explains the benefits and risks, and alternatives, to that particular adaptive intervention.  • Questions such as how much uncertainty is needed to maintain equipoise and who determines the degree of uncertainty were posed.  Recommended future directions: Developed guidance for complex clinical trial design terminology, standards for informed consent documentation for adaptive trials, and clear guidance for how researchers explain adaptive designs to facilitate better understanding among institutional research boards and participants |
| World Health Organization (8) | 2024 | Switzerland | • The WHO Secretariat conducted stakeholder consultations on the most relevant existing guidance documents for best practices regarding clinical trials and a draft of the Guidance was disseminated for public consultation; a total of 179 responses from 48 countries were received  • Additionally, the WHO Secretariat organized a consultation with private sector representatives during the 76th World Health Assembly and held an information session together feedback from Member States in September 2023.  • A global stakeholder survey was launched in August 2023, in collaboration with the WHO Collaborating Centre for research information sharing, e-learning, and capacity development, to identify barriers in conducting clinical trials and propose priority actions, with nearly 3000 responses received.  • Outcomes from the global stakeholder survey were further discussed in in-person consultations held in additional countries, attended by about 300 experts and stakeholders. | • Patient, participant and community engagement are outlined as central to trial planning and implementation phases to ensure the research meets public needs and maintains trust. Major recommendations focus on reforms that enable trials in underrepresented populations such as children, pregnant women and older adults.  • The Guidance describes how to focus trial design and oversight on scientific and ethical considerations that determine whether trials are ethical, efficient and informative, emphasizing proportionate approaches to risk. Points to a need for promoting understanding and adoption of innovative trial designs, including adaptive platform designs.  • The Guidance recommends that researchers consider the use of innovative, adaptive study designs where the trial design would decrease the study complexity and burden for participants and support generation of reliable evidence. |
| World Health Organization (46) | 2022 | Switzerland | Summary report of a two-day meeting on ethics and adaptive platform trial design in public health emergencies, after the first 2 years of the COVID-19 pandemic, organized by the World Health Organization July 18-19, 2022. | There is a notable absence of research into what participants would find beneficial to know in deciding to participate in this type of trial, including whether there should be notification of added or removed arms during the course of the study to enrolled participants, and how to acknowledge uncertainties and/or accruing knowledge during the consent process. The report calls for empirical investigation into these areas. |
| Food and Drug Administration (47) | 2023 | USA | The draft guidance document provides recommendations on the design and analysis of trials conducted under a master protocol, as well as guidance on the submission of documentation to support regulatory review. | The draft guidance specifically notes that the informed consent process and documentation should cover all treatment arms to which the participant could be randomized, as introducing treatment arms after the initial consent could reduce comparability of participants if study participants differ between those who would, for example, consent to participating in the study drug A domain from those that would consider enrolling in the domain looking at drug B. |
| Canadian Institutes for Health Research (31) | 2022 | Canada | This chapter of TCPS-2 focuses on the ethical issues involved in the design, review and conduct of clinical trials, specifically the ethical issues associated with clinical trial design, therapeutic misconception, safety, reporting new information, and registration. The emphasis in this chapter is on ethics guidance, grounded in the core principles of Respect for Persons, Concern for Welfare, and Justice. Throughout TCPS-2, the welfare of participants is noted as taking precedence over the interests of researchers, institutions, and sponsors. | There is an increased likelihood of benefit as the trial progresses with this type of design, and adaptive randomization may prioritize participants towards interventions for which unknown side effects take longer to appear. TCPS 2 further notes that while adaptive trials may require fewer participants, this can limit opportunities for targeted sub-group analyses and therefore deeper understanding of factors that contribute to participant outcomes. |
| Ong et al. (48) | 2024 | Canada | • SIMPlified LaYered consent process on recruitment of potential participants to the Staphylococcus aureus Network Adaptive Platform (SIMPLY-SNAP) is a pragmatic, multicentre, open-label, two-arm parallel-group superiority RCT, nested within the ongoing Staphylococcus aureus Network Adaptive Platform (SNAP) trial.  • Trial commenced recruitment on 28 November 2023, and will end when the target sample size of 346 accrued. • Potentially eligible participants of the SNAP trial will be randomized 1:1 to a full-length ICF or a layered consent process.  • The primary outcome is recruitment into the SNAP trial. Secondary outcomes include patient understanding of the clinical trial, patient and research staff satisfaction with the consent process, and time taken for consent. As an exploratory outcome, measures of diversity will be compared, according to the consent process randomised to. | N/A - protocol |
| Lowther & Burry (49) | 2024 | Canada | CAPTIVATE is a multi-center, open-label, parallel-group study within a trial evaluating novel consent methods for patients eligible for three large adaptive platform trials, including the Platform of Randomized Adaptive Clinical Trials in Critical Illness (PRACTICAL) trial. Patients will be randomly allocated to receive either a novel consent method using video and infographics, compared to the standard paper based informed consent method. | N/A - protocol |
| O'Grady et al. (29) | 2023 | Canada | • Patient-centred co-design study conducted to refine and test an infographic to supplement the consent documents for an ongoing, international clinical trial, the Randomized, Embedded Multi-factorial Adaptive Platform Trial for Community Acquired Pneumonia (REMAP-CAP)'s consent documents. Infographic prototypes were developed by patients, substitute decision-maker (SDMs), and researchers with lived experience in the ICU or with ICU research.  • The protocol describes the use of a two-phase exploratory sequential, mixed-methods research design. Phase 1 will consist of focus groups with ICU patients, SDMs, and research coordinators. Inductive content analysis will be used to inform infographic refinement, and pilot tested in phase 2. Phase 2 will be a prospective study within a trial at ≤ 5 REMAP-CAP sites, whereby self-reported data from patients/SDMs and research coordinators will be collected.  • The primary outcome is feasibility (eligible consent encounters, receipt of infographic, consent to follow-up, completion of follow-up surveys). Data will be integrated to understand if/how quantitative results build upon the qualitatively informed infographic. | N/A - protocol |
